# Quantitative Corticospinal Tract Assessment in Acute Intracerebral Hemorrhage

**DOI:** 10.1007/s12975-020-00850-9

**Published:** 2020-09-21

**Authors:** Bastian Volbers, Angelika Mennecke, Nicola Kästle, Hagen B. Huttner, Stefan Schwab, Manuel A. Schmidt, Tobias Engelhorn, Arnd Doerfler

**Affiliations:** 1 Department of Neurology, University of Erlangen-Nuremberg, Schwabachanlage 6, 91054 Erlangen, Germany; 2 Department of Neuroradiology, University of Erlangen-Nuremberg, Erlangen, Germany

**Keywords:** Intracerebral hemorrhage, MRI, DWI, Outcome research, All rehabilitation

## Abstract

Intracerebral hemorrhage (ICH) prognostication during the acute phase is often subjective among physicians and often affects treatment decisions. The present study explores objective imaging parameters using quantitative corticospinal tract (CST) fiber reconstruction during the acute phase of ICH and correlates these parameters with functional outcome and patient recovery. We prospectively enrolled nonsurgical spontaneous supratentorial ICH patients and obtained an MRI scan on day 5 ± 1. Q-space diffeomorphic reconstruction was performed using DSI Studio, and quantitative anisotropy (QA) was calculated. The CST was reconstructed based on QA. The dichotomized modified Rankin Scale score on day 90 (favorable outcome = 0–2) and Barthel Index (favorable recovery = 100 on day 90 or improvement between discharge and day 90 > 60%) were assessed. Thirty-three patients, median age 72 years (interquartile range (IQR) 64–83), 21 female (64%), 21 (64%) with lobar hemorrhage, median ICH volume on admission 15.0 (IQR 7.0–27.4) mL, were included. Sixteen patients (48%) had a favorable outcome and 24 (73%) had a favorable recovery. The mean number of ipsilesional reconstructed CST fiber pathways was higher in patients with favorable outcomes (153 (standard deviation (SD) 103) vs. 60 (SD 39), *p* = 0.003) and predicted outcome after adjustment (Exp(B) = 1.016 (95% CI = 1.002–1.030)). QA in the ipsilesional posterior limb of the internal capsule showed a trend towards an association with favorable outcome (Exp(B) = 1.194 (95% CI = 0.991–1.439 (adjusted))). The total (ipsilesional + contralesional) number of reconstructed fiber pathways was associated with favorable recovery (Exp(B) = 1.025 (95% CI = 1.003–1.047 (adjusted))). Quantitative tractography parameters assessed in the acute phase of ICH may represent a promising predictor of long-term outcome and recovery. This might facilitate prognostic evaluation and organization of rehabilitation.

**Electronic supplementary material:**

The online version of this article (10.1007/s12975-020-00850-9) contains supplementary material, which is available to authorized users.

## Introduction

Many approaches have been proposed to improve the evaluation of long-term functional outcome in the acute phase of intracerebral hemorrhage (ICH), including clinical parameters [1] or clinical grading scales [2]. However, physicians still vary substantially in performing ICH prognosis estimates and consequently in developing treatment recommendations [3] with possible effects on decision-making, rehabilitation and functional outcome.

Functional outcome after ICH is highly dependent on the degree of motor recovery [4, 5]. Here, corticospinal tract (CST) integrity represents not only a measure for residual motor function but also for motor function recovery [6]. Advanced postprocessing methods such as diffusion tensor imaging (DTI) using diffusion-weighted magnetic resonance imaging (MRI) [7], currently the only method capable of mapping the fiber architecture of nervous tissue in vivo [8], allow pathway reconstruction, which could be shown to reliably visualize the CST [9]. Regarding outcome prediction in ICH patients two major issues arise: First, little data exist regarding the association of quantitative tractography assessment with outcome in ICH. Most studies either focused on the analysis of fractional anisotropy (FA), a scalar measure describing the degree of water diffusion hindrance and/or restriction in particular directions, either in ischemic stroke [10] or ICH [11–15] or performed a qualitative CST pathway assessment (absent vs. incomplete vs. complete reconstruction) [16–19] based on FA-related reconstruction. Second, as FA may be biased by multiple parameters, such as crossing fibers or partial volume effects, it may yield limited results when used for tractography [8]. Here, q-space diffeomorphic reconstruction (QSDR), a novel method derived from q-ball imaging [20, 21] resulting in quantitative anisotropy (QA), was proposed to improve the accuracy of quantitative tractography assessment [22].

## Methods

### Patient Selection

Patients were prospectively screened for eligibility between 08/2012 and 08/2015. Inclusion criteria were spontaneous supratentorial hemorrhage in patients aged > 18 years. Exclusion criteria included secondary ICH due to trauma, AV-malformation, tumor or sinus thrombosis, surgical evacuation or trepanation, additional subarachnoid hemorrhage or sub- or epidural hematoma, contraindication against MRI examination or contrast enhancer, pregnancy, an unstable cardiopulmonary condition, early limitation of care or stroke or ICH in the patient’s history.

### Patients’ Treatment

All patients were treated according to national guidelines, the European Stroke Initiative guidelines for the monitoring and treatment of ICH [23] and institutional standard operation procedures. Patients received physiotherapy within 24 h after admission and during the in-hospital stay. A rehabilitative treatment after discharge was organized for each patient according to the Barthel Index (BI) at discharge.

### MR Diffusion Imaging

MRI was performed on day 5 ± 1 after admission to minimize bias by ensuring sufficient imaging comparability since MRI signals in ICH may change in a time-dependent manner during the acute phase. The diffusion-weighted images were acquired on a 1.5 T scanner (Magnetom Aera, Siemens Healthcare, Erlangen, Germany) with a 20-channel phased-array head and neck coil. DTI was performed in the axial plane with an in-plane resolution of 1.8 mm and 5 mm slice thickness using a single-shot, spin echo, echo planar imaging (EPI) diffusion tensor sequence (TR = 4000 ms, TE = 83 ms, FoV = 230 × 230 mm^2^, acquisition matrix size = 128 × 128, number of signal averages = 2, GRAPPA factor = 2, partial Fourier acquisition = 75%). Diffusion weighting was carried out with a maximal *b*-factor of 1000 s/mm^2^ along 20 icosahedral diffusion directions supplemented by one scan with *b* = 0 s/mm^2^.

### Assessment of Artifacts and Data Processing

**Fig. 1:**
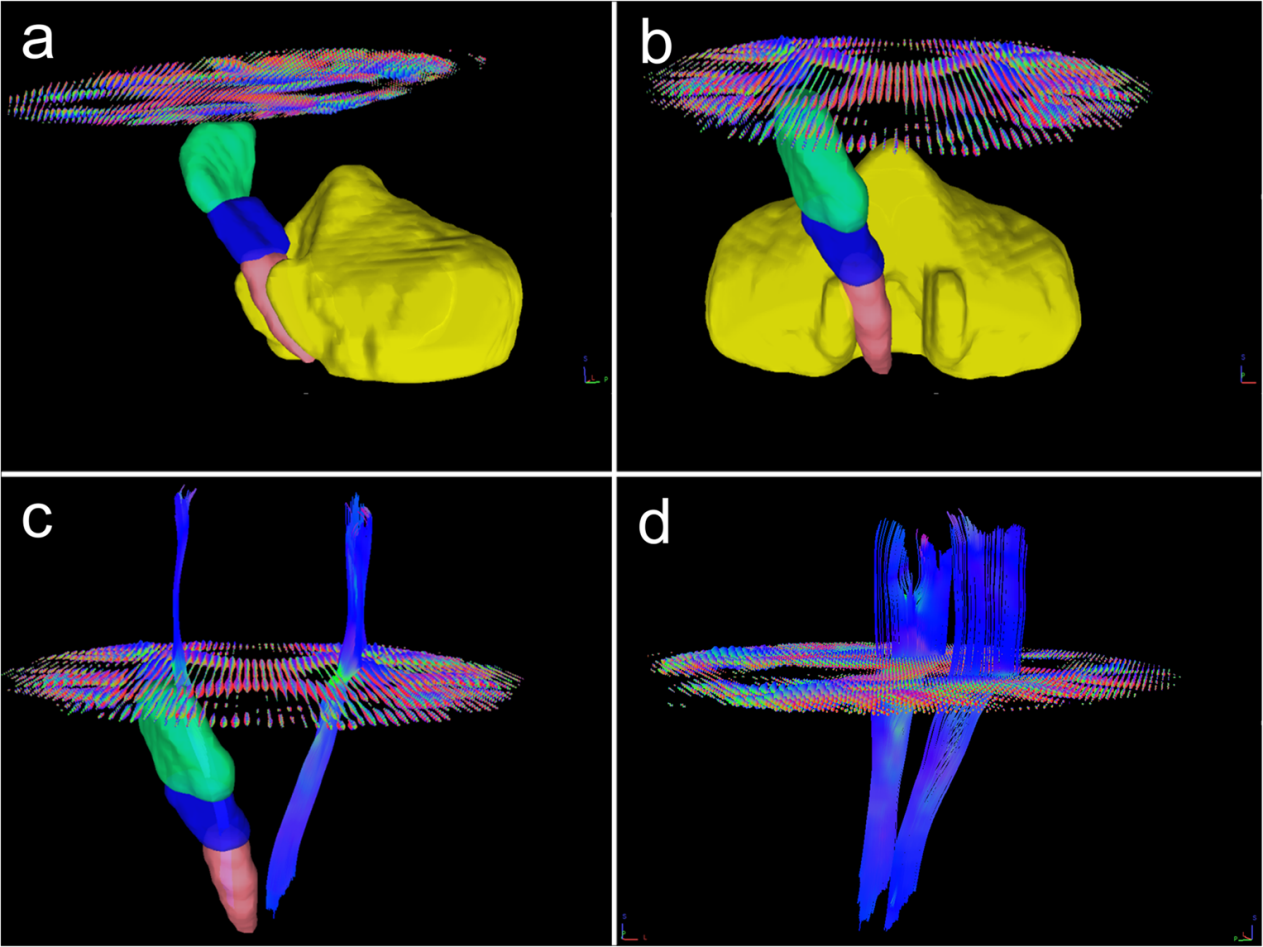
Tractography and reconstructed fiber pathways including an axial slice of the orientation density function (ODF). **a**, **b** Region of interest (ROI)/seeding region: green: posterior limb of internal capsule (PLIC), dark blue: cerebral peduncle (= ROI), red: corticospinal tract (= seeding region); region of avoidance: yellow: cerebellum. **a** Lateral view; **b** anterior-posterior view. **c**, **d** Reconstructed fiber pathways (corticospinal tract). **c** Anterior-posterior view including the seeding, the ROI, and the PLIC region on the right side; **d** lateral view

Mean FA and QA values were calculated for the CST region, the cerebral peduncle (CP) region and the posterior limb of the internal capsule (PLIC) region according to the John Hopkins University (JHU) white matter atlas labels (1 mm) [24]. The number of the reconstructed fiber pathways was calculated by DSI Studio (see Fig. 1 for a depiction of the seeding regions, region of interest (ROI) and region of avoidance (ROA) as well as a representative fiber pathway reconstruction).

For better comparability with pre-existing results, a tract-based statistical (TBSS) analysis of fractional anisotropy as well as FA-based deterministic fiber tracking (diffusion tensor imaging, DTI) was additionally performed using the same tracking parameters except that repeated tracts (duplicates) were not removed. Detailed information is given in Online Resource 1 and Fig. 1.

### Outcome Parameters

The modified Rankin Scale score and Barthel Index were obtained on day 90 by a trained neurologist blinded to the patient’s admission diagnosis via telephone interview. The modified Rankin Scale score was dichotomized into favorable outcomes (0–2) and poor outcomes (3–6). A favorable recovery was defined as a BI of 100 on day 90 or a BI improvement of > 60% (concept of proportional recovery) between discharge and day 90 [25]. We also linearly correlated DTI measures with the NIHSS motor domain score (=sum of upper and lower motor NIHSS scores) on admission and day 5 and dichotomized them according to the median split method (favorable: ≤ median). The number of reconstructed fiber pathways as well as the mean fractional anisotropy (FA) and quantitative anisotropy (QA) within the regions of interest (CST, CP and PLIC) were analyzed. Regarding recovery analysis, we additionally calculated the asymmetry index of PLIC integrity as described by Stinear et al. using both FA and QA values (FA/QA_contralesional_ − FA/QA_ipsilesional_) / (FA/QA_contralesional_ + FA/QA_ipsilesional_) [26]. Here, positive values indicate reduced FA/QA in the affected PLIC while 0 corresponds to symmetrical FA/QA in both PLICs.

### Statistics

Statistical analyses were performed using the IBM® SPSS® Statistics 21 software package (IBM-Corporation, Armonk, NY). Data are presented as the mean and standard deviation (SD), median and interquartile range (IQR) or *n* (%) as appropriate. Normally distributed (according to the Kolmogorov-Smirnov test) interval data were compared between outcome groups using independent t-tests; other interval and ordinal data were compared using the Wilcoxon rank-sum test. The *χ*^2^/Fisher’s exact test was used to analyze differences in nominal data between outcome groups. Diffusion imaging parameters associated with outcome or recovery in univariable analyses were further analyzed adjusted for all other meaningful variables with at least a trend towards an association (*p* value < 0.1) [27] using multivariable logistic regression (forced inclusion and backward LR). Linear regression was used to correlate NIHSS (motor domain) scores with the number of ipsilesional reconstructed fiber pathways. Receiver operating characteristic curve analyses including the Youden Index were performed to evaluate the predictive value of selected diffusion imaging parameters and to establish cutoff points for outcome and recovery prediction. A two sided *p* value of < 0.05 was defined as significant.

## Results

### Patient Characteristics

**Fig. 2:**
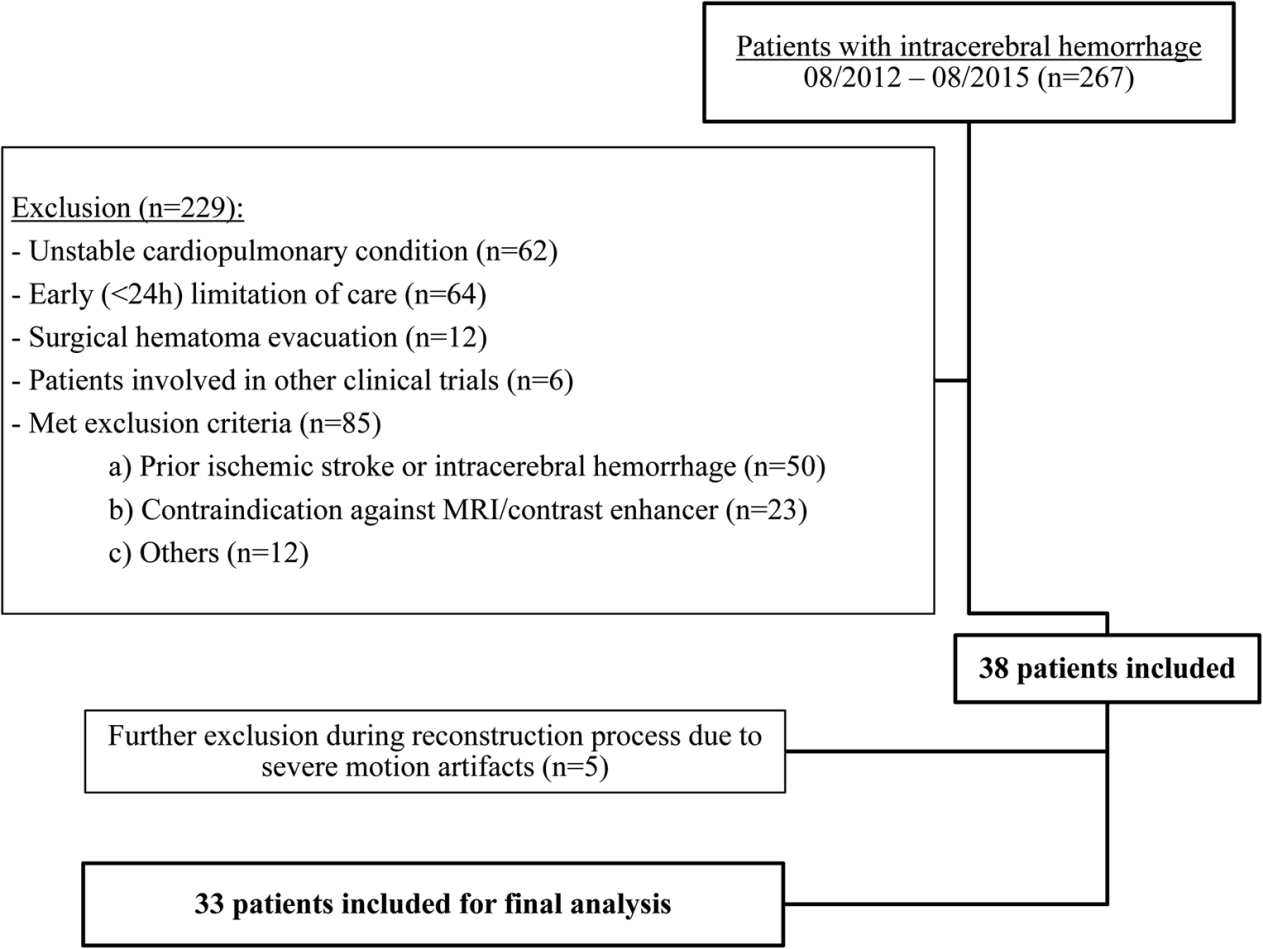
Flowchart of included and excluded patients. MRI, magnetic resonance imaging

A total of 267 patients were screened. Please see Fig. 2 for a flowchart of the inclusion and exclusion process. The median age was 72 years (IQR 64–83), and the median hematoma volume on admission was 15.0 mL (IQR 7.0–27.4), with 12 (36%) hematomas located in the basal ganglia. Sixteen (48%) patients showed a favorable day 90 outcome. Twenty-four patients (73%) showed a favorable recovery. Patients with a favorable day 90 outcome and those with a favorable recovery were younger and presented with milder clinical symptoms on admission. Furthermore, patients with a favorable recovery showed a lower baseline mRS before ICH onset than patients with poor recovery. Other clinical characteristics were equally distributed between both the outcome and recovery groups (see Tables 1 and 2).

**Table 1:** Clinical characteristics of patients with favorable and poor outcomes. Data are given as the mean and standard deviation (SD), median and interquartile range (IQR), or number and percentage (%) as appropriate; *NIHSS*, National Institutes of Health Stroke Scale score; *GCS*, Glasgow Coma Scale; *mRS*, modified Rankin Scale score, *baseline mRS*, mRS during the week before symptom onset; *VKA*, vitamin K antagonist; *DOAC*, direct oral anticoagulant

	Favorable outcome (mRS 0–2); *n* = 16)	Poor outcome (mRS 3–6; *n* = 17)	*p* value
Age (median years (IQR))	70 (59–74)	76 (68–85)	0.016^†^
Female sex (*n* (%))	8 (50)	13 (76)	0.157*
NIHSS on admission (median (IQR))	2 (1–4.75)	9 (2–14.5)	0.021^†^
Right-handed patient (*n* (%))	14 (88)	15 (88)	0.948*
Baseline mRS (median (IQR))	0 (0)	0 (0–0.5)	0.402^†^
Hypertension (*n* (%))	14 (88)	15 (88)	0.948*
Diabetes mellitus (*n* (%))	2 (13)	6 (36)	0.127*
Renal insufficiency (*n* (%))	3 (19)	2 (12)	0.576*
Atrial fibrillation (*n* (%))	4 (25)	3 (18)	0.606*
Platelet aggregation inhibitors/oral anticoagulation (*n* (%))	4 (25)	7 (41)	0.270*
Location basal ganglia (*n* (%))	4 (25)	8 (47)	0.188*
Location lobar (*n* (%))	12 (75)	9 (53)	0.188*
Hematoma on right side (*n* (%))	5 (31)	9 (53)	0.208*
Intraventricular hemorrhage (*n* (%))	3 (19)	5 (29)	0.475*
Hematoma volume on admission (median mL (IQR))	12.3 (5.7–20.4)	18.0 (7.1–28.6)	0.471^†^
Length of stay (median days (IQR))	11 (7–12.75)	9 (7.5–12.5)	0.999^†^

*χ^2^/Fisher’s exact test when necessary

^†^Wilcoxon rank-sum test

**Table 2:** Characteristics of patients with favorable and poor recovery. Data are given as median and interquartile range (IQR) or number and percentage (%) as appropriate; *BI*, Barthel Index; *NIHSS*, National Institutes of Health Stroke Scale score; *baseline mRS*, modified Rankin Scale score during the week before symptom onset; *QA*, quantitative anisotropy; *FA*, fractional anisotropy; *PLIC*, posterior limb of internal capsule

	Favorable recovery (BI improvement between discharge and day 90 > 60% or day 90 BI = 100, *n* = 24)	Poor recovery (BI improvement between discharge and day 90 < 60%, *n* = 9)	*p* value
Age (median years (IQR))	70 (59–75)	84 (73–86)	0.002^†^
Female sex (*n* (%))	13 (54)	8 (89)	0.107*
Baseline mRS (median (IQR))	0 (0)	0 (0–2)	0.043^†^
Hematoma volume on admission (median mL (IQR))	12.0 (5.5–20.4)	24.1 (12.8–29.2)	0.102^†^
Intraventricular hemorrhage (*n* (%))	4 (17)	4 (44)	0.170*
Location basal ganglia (*n* (%))	9 (38)	3 (33)	0.999*
NIHSS on admission (median (IQR))	2 (1–8)	14 (5–15)	0.008^†^
BI on discharge (median (IQR))	90 (50–100)	15 (0–35)	0.003^†^
QA asymmetry index of PLIC (median (IQR))	− 0.036 (− 0.06 to − 0.004)	0.046 (0.021 to 0.098)	0.001^†^
FA asymmetry index of PLIC (median (IQR))	− 0.005 (− 0.033 to 0.036)	0.076 (−0.001 to 0.1)	0.049^†^
Number of ipsilesional reconstructed fiber pathways (QA based) (median (IQR))	102 (48–192)	47 (16–95)	0.060^†^
Number of contralesional reconstructed fiber pathways (QA based) (median (IQR))	116 (61–174)	15 (1–53)	< 0.0001^†^
Number of total reconstructed fiber pathways (ipsilesional + contralesional (QA based) (median (IQR))	224 (122–328)	47 (31–130)	0.001^†^

*χ^2^/Fisher’s exact test when necessary

^†^Wilcoxon rank-sum test

### Fractional and Quantitative Anisotropy

**Fig. 3:**
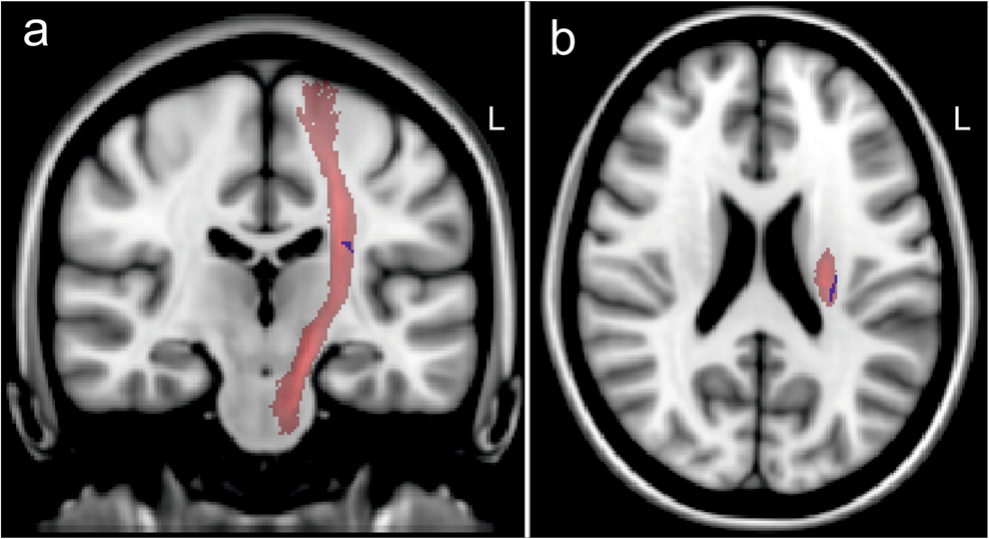
Tract-based spatial statistics (TBSS). Decreased fractional anisotropy (FA) of the corticospinal tract (CST) of the affected hemisphere (left side (L); data sets of patients with right hemisphere hemorrhage have been flipped right-left). Red: CST-mask (JHU White Matter Tractography Atlas). Blue: clusters of voxels with decreased FA (*p* = 0.22, corrected). **a** Coronal view. **b** Axial view. Data superimposed on the MNI152 T1-brain mask for anatomical orientation

TBSS showed decreased FA of the ipsilesional CST (periventricular; approximately PLIC level) without reaching statistical significance (see Fig. 3). The median FA values of the PLIC according to the JHU White Matter Atlas were similar (ipsilesional = 0.46 (IQR 0.39–0.50), contralesional = 0.48 (IQR 0.45–0.51), *p* = 0.108).

#### Association with Recovery

The FA or QA values did not differ for any ROI between patients with favorable and poor recovery (data not shown). The FA and QA asymmetry indexes (PLIC) differed between patients with favorable and poor recovery (see Table 2). After adjustment for age, baseline mRS and NIHSS scores on admission (forced inclusion), the QA asymmetry index remained associated with a favorable recovery (adjusted odds ratio (OR) < 0.001 (95% CI = 0–0.150)).

#### Association with Outcome

Mean FA values within the prespecified ROI did not differ between patients with favorable and poor outcomes (see Table 3). QA values were higher in the ipsilesional PLIC and ipsilesional CP in patients with favorable outcomes than in patients with poor outcomes, while there was no difference in ipsilesional CST levels (see Table 3). Contralesional QA values did not differ between both outcome groups for any ROI. After adjustment for age and NIHSS score on admission (forced inclusion), there was a trend towards an association of mean ipsilesional QA (PLIC (OR = 1.194 (95% CI = 0.991–1.439)) and CP (OR = 1.218 (95% CI = 0.964–1.538))) with favorable outcome. Patients with poor outcomes showed lower ipsilesional than contralesional QA values while patients with favorable outcomes had higher ipsilesional QA values (see Table 3); however, these differences were not significant (PLIC: poor outcome *p* = 0.344, favorable outcome *p* = 0.327, data for other ROIs not shown).

**Table 3:** Fractional and quantitative anisotropy and characteristics of reconstructed fiber pathways in patients with favorable and poor outcomes. Data are given as the mean and standard deviation (SD) or median and interquartile range (IQR) as appropriate. mRS, modified Rankin Scale score; FA, fractional anisotropy; QA, quantitative anisotropy; CST, corticospinal tract region^1^; CP, cerebral peduncle region^1^; PLIC, posterior limb of internal capsule region^1^

	Favorable outcome (mRS 0–2; *n* = 16)	Poor outcome (mRS 3–6; *n* = 17)	*p* value
FA CST ipsilesional	0.37 (SD 0.06)	0.35 (SD 0.05)	0.200^†^
FA CST contralesional	0.37 (SD 0.05)	0.37 (SD 0.05)	0.929^†^
FA CP ipsilesional	0.47 (SD 0.07)	0.43 (SD 0.05)	0.109^†^
FA CP contralesional	0.46 (SD 0.04)	0.45 (SD 0.05)	0.306^†^
FA PLIC ipsilesional	0.49 (IQR 0.45–0.53)	0.42 (IQR 0.38–0.49)	0.063^*^
FA PLIC contralesional	0.48 (SD 0.04)	0.46 (SD 0.04)	0.306^†^
QA CST ipsilesional	9.6 (IQR 6.2–11.7)	6.9 (IQR 5.2–7.7)	0.063*
QA CST contralesional	8.6 (IQR 6.1–12.0)	6.8 (IQR 5.6–7.8)	0.102*
QA CP ipsilesional	11.1 (IQR 8.5–13.8)	7.2 (IQR 5.8–10.0)	0.028*
QA CP contralesional	10.2 (IQR 7.4–13.8)	8.2 (IQR 6.2–9.9)	0.127*
QA PLIC ipsilesional	18.9 (IQR 16.2–23.3)	14.6 (IQR 11.7–17.7)	0.015*
QA PLIC contralesional	18.0 (IQR 15.0–21.3)	15.8 (IQR 13.0–18.7)	0.363*
Number of ipsilesional reconstructed fiber pathways (QA based)	153.3 (SD 102.8)	59.8 (SD 38.6)	0.003^†^
Number of contralesional reconstructed fiber pathways (QA based)	99.8 (SD 59.1)	57.4 (SD 61.9)	0.053^†^
Number of ipsilesional reconstructed fiber pathways (FA based)	4036 (SD 3606)	1733 (SD 1427)	0.027^†^
Number of contralesional reconstructed fiber pathways (FA based)	6129 (SD 5212)	3145 (SD 2560)	0.051^†^

^1^According to JHU White Matter Labels

*Wilcoxon rank-sum test

^†^Independent *t* test

### Quantitative Tractography Assessment

#### Association with Motor Function

Motor NIHSS score on day 5 was linearly associated with the number of ipsilesional reconstructed fiber pathways (R^2^ = 0.142, *p* = 0.018). The median motor NIHSS score on both admission and day 5 was 1. Patients with a favorable motor NIHSS score on day 5 (≤ 1, *n* = 17) showed more ipsilesional reconstructed fiber pathways than patients with a poor motor NIHSS score (day 5: 142 (SD 93) and 66 (SD 68), *p* = 0.011; on admission (*n* = 17): 153 (SD 95) and 54 (SD 42), respectively, *p* = 0.001). The number of contralesional reconstructed fiber pathways did not differ between the motor groups (day 5: favorable: 88 (SD 60), poor: 67 (SD 67), *p* = 0.356; on admission: 87 (SD 60) and 68 (SD 68), *p* = 0.394).

#### Association with Recovery

Multivariable logistic backward LR regression adjusted for age and baseline mRS and NIHSS scores on admission revealed an independent association of complete number (ipsi- and contralesional) of reconstructed fiber pathways with favorable recovery (OR = 1.025 (95% CI = 1.003–1.047)). Ipsilesional reconstructed fiber pathways showed a trend towards an association with favorable recovery (adjusted OR = 1.017 (95% CI = 0.998–1.035)). In a subset of patients with low BI at discharge (< 50P = severely affected; *n* = 14), the median number of contralesional reconstructed fiber pathways was higher in patients with a proportional recovery > 60% (*n* = 6) than in patients with a proportional recovery < 60% (*n* = 8; 113 (IQR 54–184) and 11 (IQR 1–21), *p* = 0.005), while ipsilesional reconstructed fiber pathways did not differ (48 (IQR 15–118) and 37 (IQR 14–84), *p* = 0.950).

#### Association with Functional Outcome

The number of ipsilesional reconstructed fiber pathways (QA based) was higher in patients with favorable outcomes than in patients with poor outcomes. The number of contralesional reconstructed fiber pathways showed an increasing trend in patients with favorable outcomes (see Table 3). Thus, we also analyzed contralesional pathways with multivariable analysis, which did not reveal an independent association with outcome (OR = 1.016 (95% CI = 0.995–1.025)). After adjustment for age and NIHSS score on admission (forced inclusion; logistic regression), the number of ipsilesional reconstructed fiber pathways remained an independent predictor of functional outcome (OR = 1.016 (95% CI = 1.002–1.030)). The number of ipsilesional reconstructed fiber pathways based on FA also differed between both outcome groups (see Table 3). After adjustment, quantitative assessment of FA-based tractography (DTI) did not predict outcome (OR = 1.000 (95% CI = 1.000–1.001)).

### Clinical Significance and Predictive Value of Diffusion Imaging Characteristics

**Fig. 4:**
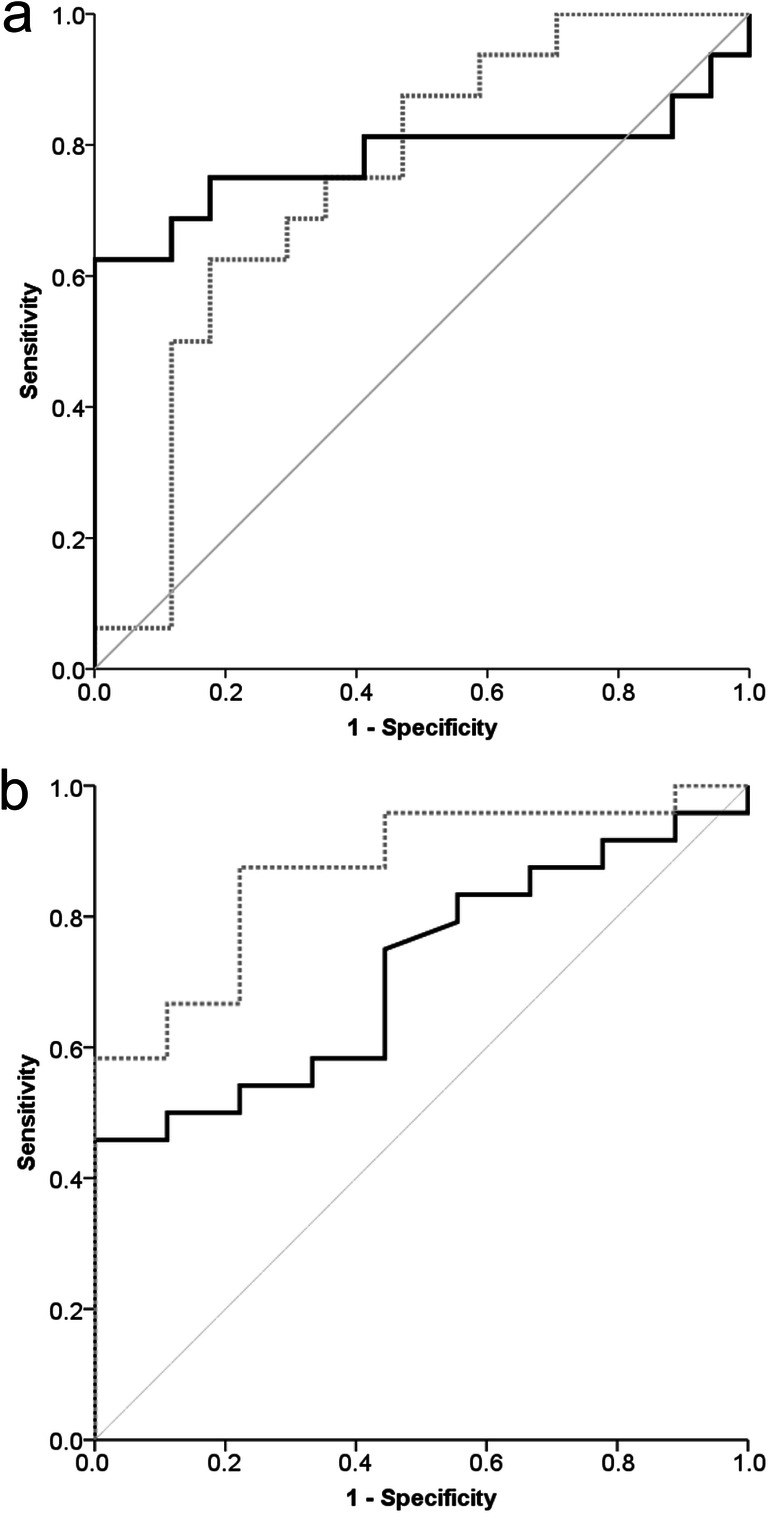
Receiver operating characteristic curves for prediction of outcome and recovery. **a** Association of number of ipsilesional reconstructed fiber pathways (black line) and mean quantitative anisotropy (QA) of the ipsilesional posterior limb of the internal capsule (PLIC, dotted gray line) with favorable outcome. Receiver operating characteristic curves for the prediction of favorable day 90 outcome (mRS score 0–2). Gray line = reference line. Ipsilesional fiber number: AUC = 0.779 (95% CI = 0.597–0.962). Mean QA ipsilesional PLIC: AUC = 0.746 (95% CI = 0.574–0.919). **b** Association of number of total (ipsilesional + contralesional, gray dotted line) and ipsilesional (black line) reconstructed fiber pathways with favorable recovery (BI improvement between discharge and day 90 > 60% or day 90 BI = 100). Ipsilesional pathway number: AUC = 0.715 (95% CI = 0.537–0.893). Complete pathway number: AUC = 0.87 (95% CI = 0.744–0.997).

These findings were supported by receiver operating characteristic analyses exploring the association of the number of ipsilesional reconstructed fiber pathways (AUC = 0.779 (95% CI = 0.597–0.962)) and mean QA values in the ipsilesional PLIC (AUC = 0.746 (95% CI = 0.574–0.919)) with favorable outcome (Fig. 4). A total of 140 ipsilesional reconstructed fiber pathways were detected as an optimal cutoff for predicting a favorable outcome (sensitivity = 0.625, specificity = 1.0, Youden Index = 0.625). Regarding favorable recovery, the total number of reconstructed fiber pathways showed good predictive value (AUC = 0.87 (95% CI = 0.744–0.997)) with an optimal cutoff of 106 (Youden Index = 0.653, sensitivity = 0.875, specificity = 0.778).

## Discussion

To date, DTI research in ICH patients has focused mainly on the predictive value of FA and a rather qualitative DTT approach. Decreased FA values have been found in the ipsilesional cerebral peduncle [12, 13, 18, 28], CST [10, 29] and pons and have been shown to be associated with poor outcome. However, this was not consistently shown in all studies [30]. Given the physical properties of FA and the abovementioned results, FA decrease is interpreted as a marker of progressive Wallerian degeneration [31] and decreased CST integrity after ICH or ischemic stroke. However, different FA values in patients with favorable and poor outcomes have been reported, which may partly be explained by differences in outcome measures and different time points of MRI scanning. Our data show a trend towards reduced FA values in the ipsilesional PLIC of ICH patients with poor outcome but not in regions more distant from the ICH location like the CST region or when analyzing the FA ratio (data not shown) between the affected and unaffected sides in any location. Additionally, an unadjusted FA-based asymmetry index of PLIC integrity [26] is associated with recovery. Thus, the general meaningfulness of FA in ICH outcome research is somehow confirmed. Again, methods, outcome measures and especially early MRI performance in our study differed from existing studies.

Another approach for ICH patients is qualitative DTT assessment. It has been shown that interrupted or absent ipsilesional reconstructed CST streamlines [16, 17] are associated with poor outcome in ICH patients. A quantitative analysis was not conducted in those studies. Bigourdan and colleagues performed quantitative DTT analysis within 72 h after symptom onset of ischemic stroke and found that the fiber count ratio, defined as the ipsilesional streamline number normalized to the unaffected side, independently predicted 1-year motor outcome assessed by the Fugl-Meyer score [5]. ICH patients were not included. We found an association of ipsilesional FA-based streamline count with outcome assessed on day 90 using the modified Rankin scale in the unadjusted analysis but not after adjustment. We did not calculate the ratio between the affected and unaffected sides, as this procedure may be problematic due to the complex interaction with known contralesional changes after ischemic stroke or ICH [19] and with patient status before the current ICH.

In general, FA-related research is associated with possible limitations. It is hypothesized that FA correlates with white matter fiber density, with high FA values representing a high proportion of diffusion along a certain direction due to a high fiber density as a sort of natural border hindering or restricting free diffusion of water [8, 31]. However, the interpretation of FA values and tractography based on it may be limited, especially when voxels containing more than one fiber population or crossing fibers are involved, which is the general rule within the brain [8]. Furthermore, the diffusion tensor model underlying the FA calculation assumes anisotropic Gaussian diffusion, which does not account for more complex directional dependencies or diffusion changes due to the presence of different compartments [8]. Additionally, partial volume effects or temperature may affect FA values. Performing quantitative streamline analysis information on spin density may be needed, which FA does not contain.

Here, several strengths of our study might add further insights to existing knowledge:

We performed a standardized MRI assessment in the acute phase of ICH on day 5 ± 1. Outcome was assessed by a trained neurologist on day 90 using the modified Rankin scale score as an established outcome parameter and measure for disability in stroke research [32]. We applied motion and eddy-current correction including correction of the encoding vectors [8] to our data as well as a reconstruction method called q-space diffeomorphic reconstruction, which handles crossed-fiber issues well [20, 22]. Using QSDR, assumption-free modeling is possible, resulting in a measure called quantitative anisotropy (QA), which is defined as the number of spins that undergo diffusion along any given fiber orientation [20, 33]. QA is derived from an orientation density function (ODF)-based index scaled with the spin density function (SDF) [22]. The ODF [21] is thought to account for multiple fiber orientations within each voxel, and the SDF is defined as the number of spins that undergo diffusion in different orientations [33]. It can be shown that deterministic fiber tracking is improved by using QA-aided tractography [22]. Furthermore, QSDR data were transformed into MNI space, which allowed the use of predefined ROIs, resulting in a more valid and comparable analysis across subjects.

Our results suggest that the presence of ICH may be related to changes within the CST, which are associated with patient outcome and recovery and may be visualized and quantified using our algorithm. Whether the number of “real” CST neurons is truly reduced by hematoma-caused disruption of brain tissue or already initiated secondary Wallerian degeneration cannot be concluded from DTI-derived data [8].

The fact that reduced streamlines on the unaffected contralateral side also showed a trend towards an association with poor outcome may be interpreted as a hint that the pre-existing patient status also plays a role in long-term outcome. Baseline functional status did not differ between outcome groups, while it did differ between recovery groups. Accordingly, the contralesional CST seems even more important when analyzing recovery, especially in severely affected patients with severe ipsilesional damage. Other studies have shown that contralesional diffusion tensor-related CST measures might also have an association with outcome [34]. It remains unclear whether these measures reflect a pre-existing condition or a contralesional (mal-)adaption in hemorrhagic stroke patients [19]. Furthermore, sensory loss may also be associated with outcome and recovery [35]. Since the PLIC also contains sensory fibers, including fibers of the posterior thalamic radiation [36], PLIC-related anisotropy assessment may also reflect lesions affecting the sensory nervous system. In our study, patients with favorable outcomes and recovery also had better sensory function as quantified using the sensory item of the NIHSS. However, since the focus of the present manuscript was motor function, we did not report these data or assess sensory fiber pathways. More complex modeling is needed to analyze these interactions [19].

There are several limitations to our study. First, a small single-center cohort was analyzed, which may limit the generalizability of our data. The majority of screened patients were excluded due to our prespecified criteria, including pre-existing disability, a surgical intervention, an existing do-not-resuscitate order or a prior ischemic stroke/intracerebral hemorrhage in the patients’ history. However, due to strict inclusion criteria and the prospective design of the study, we obtained a robust data set. The restricted availability of MR imaging, including tractography analysis tools, may limit the widespread use of this technique at this time. Despite motion correction, our data processing method is susceptible to movement artifacts. Due to its low sensitivity to motion artifact, we used single-shot echo planar imaging (ss-EPI) as a widely established standard for DTI. However, this technique is prone to susceptibility artifacts [37] and may be associated with image distortion and spatial voxel displacement [38]. Parallel imaging, as used in the present study, has been shown to improve the signal-to-noise ratio and image distortion due to susceptibility artifacts [39, 40]. Other workgroups further demonstrated that susceptibility-related effects on pathway reconstruction are rather small even in hematoma-adherent regions [28]. Previous research correlated reconstructed CST pathways with motor scores such as the Fugl-Meyer-Score. We decided to obtain the modified Rankin Scale score on day 90 with a focus on ADLs, relying mainly on motor function as an established outcome measure in stroke research. Additionally, we gave results regarding the motor domain of the NIHSS. The reconstruction of continuous fiber pathways may be influenced by, e.g., curvature, length or width of axons [8]. Despite the use of an advanced and sophisticated algorithm, these factors might also pose a possible bias in our results. Moreover, the QSDR transformation may not have worked accurately in patients with large hematoma volumes due to structural differences from the reference MNI template. Thus, non-sense reconstructed fiber pathways were removed manually, which may have influenced our results. However, fibers were removed following consensus from two readers. QA thresholds used for fiber tracking were detected automatically. This procedure was necessary, as QA values are not comparable across individual patients. No longitudinal assessment of MRI scans or structural connectivity analysis was performed. Location-dependent outcome analysis showed comparable results regarding ipsi- and contralesional fiber pathways and QA values at the PLIC level, even if, due to the small cohort size, the results did not differ significantly in deep ICH (data not shown). Larger cohorts are necessary to assess location-dependent differences. We did not assess the efficacy of any rehabilitative treatment in relation to any outcome prognostication or analyze interactions with an additional sensory loss or associations with lesions affecting sensory fiber pathways.

## Summary

We showed an independent association of quantitative tractography analysis performed in the acute phase of ICH patients with long-term outcome and with recovery. In combination with other established predictive factors, quantitative tractography might improve the prognostic dilemma in ICH patients. Prospective intervention studies are needed to examine the association of rehabilitation programs with outcome, especially in patients at high risk for poor outcome and recovery.

## Electronic Supplementary Material

ESM 1 (PDF 124 kb)

## Data Availability

The data that support the findings of this study are available from the corresponding author upon reasonable request.
