# Supplementary material for: Quantitative Corticospinal Tract Assessment in Acute Intracerebral Hemorrhage
Source: Transl Stroke Res. 2020 Sep 21;12(4):540–9. doi: 10.1007/s12975-020-00850-9 (PMC8213667; doi:10.1007/s12975-020-00850-9)
Supplement: Supplementary file 1 — (PDF 124 kb) [file 12975_2020_850_MOESM1_ESM.pdf]

SUPPLEMENTAL MATERIAL
Online Supplement

Quantitative Corticospinal Tract Assessment in Acute Intracerebral Hemorrhage

Bastian Volbers[1,2], MD, Angelika Mennecke[2], Dipl.-Phys, Nicola Kästle[2], MD, Hagen B. Huttner[1], MD, Stefan Schwab[1], PhD, Manuel A. Schmidt[2], MD, Tobias Engelhorn[2], MD, Arnd Doerfler[2], MD

[1]Department of Neurology, University of Erlangen-Nuremberg, Erlangen, Germany
[2]Department of Neuroradiology, University of Erlangen-Nuremberg, Erlangen, Germany

Supplemental Methods

Assessment of artifacts
We corrected distortions induced by eddy currents and head motion by performing rigid registration of diffusion weighted images to respective b0 images using the freely available FMRIB Software Library (FSL, https://fsl.fmrib.ox.ac.uk/fsl/) toolbox[1] including the "eddy_correct" tool[2] and MATLAB (R2015b, MathWorks, Natick, Massachusetts, USA). The direction table was updated with the estimated motion matrices. We used Generalized Autocalibrating Partially Parallel Acquisitions (GRAPPA; Magnetom Aera, Siemens Healthcare, Erlangen, Germany) to implement parallel imaging at lower field strength (1.5T) to minimize the distortion generated by susceptibility artifacts[3, 4].

Data processing
For further data procession including tractography we used the freely available tractography software tool DSI studio (http://dsi-studio.labsolver.org/), a reliable and established software tool already used in more than 550 peer-reviewed publications. Diffusion data were reconstructed into the Montreal Neurological Institute (MNI; ICBM 152 atlas 2009a NLIN asymmetric template; http://www.bic.mni.mcgill.ca/ServicesAtlases/ICBM152NLin2009[5, 6]) space using q-space diffeomorphic reconstruction (QSDR)[7], a novel method also validated using phantom studies[8], to obtain the spin distribution function[9] . Calculated out of the spin density function, the spin distribution function is an orientation distribution function (ODF) of the spin quantity. A diffusion sampling length ratio of 1.25 was used, the output resolution was 2 mm. Based on the spin distribution function, the index quantitative anisotropy (QA) was calculated, which is defined as the amount of anisotropic spins that diffuse along a certain fiber orientation[9, 8]. A deterministic fiber tracking algorithm[8] based on QA was applied. An automated seeding region was placed in the corticospinal tract at pons level and an automated region of interest (ROI) in the cerebral peduncle according to the white matter (WM) John Hopkins University Atlas JHU-ICBM-labels-1mm (see figure 1)[10]. A region of avoidance (ROA) was placed in the cerebellum according to the Montreal neurological institute (MNI) template to avoid inclusion of cerebellar pathways (see figure 1). Angular threshold was 60 degrees, step size was 1 mm. The anisotropy threshold was determined automatically by DSI Studio. Tracks with a length less than 100 mm were discarded. A total of 750000 seeds were placed automatically at random within the above mentioned seeding region. The software tool reconstructed all pathways passing both a specific seeding point as well as the region of interest. All pathways passing the region of avoidance were not reconstructed. Replicated tracts (duplicates) were discarded automatically. Remaining tracts were then screened by two readers for nonsense reconstructed fiber pathways, which were removed manually after consensus of both readers had been achieved.

For voxelwise tract-based statistical (TBSS) analysis of fractional anisotropy, FA data was processed (registration to a study-specific template) using the freely available FSL toolbox (https://fsl.fmrib.ox.ac.uk/fsl/)[1]. We used the same methodology as the one employed in a previous study[11]: Data sets of patients with right hemisphere hemorrhage were right-left flipped, so that in all datasets hemorrhage was located on the left hemisphere. Then, a symmetric mean-FA image and derived skeleton was calculated, one that is restricted to those (residual) tract structures that are present on both hemispheres. Next, the 4D pre-aligned FA data was projected onto the symmetrized skeleton and was left-right tested using the randomize option.

Supplemental References:

1. Jenkinson M, Beckmann CF, Behrens TE, Woolrich MW, Smith SM. Fsl. Neuroimage. 2012;62(2):782-90. doi:10.1016/j.neuroimage.2011.09.015.
2. Smith SM, Jenkinson M, Woolrich MW, Beckmann CF, Behrens TE, Johansen-Berg H et al. Advances in functional and structural MR image analysis and implementation as FSL. Neuroimage. 2004;23 Suppl 1:S208-19. doi:10.1016/j.neuroimage.2004.07.051.
3. Griswold MA, Jakob PM, Heidemann RM, Nittka M, Jellus V, Wang J et al. Generalized autocalibrating partially parallel acquisitions (GRAPPA). Magnetic Resonance in Medicine. 2002;47(6):1202-10. doi:10.1002/mrm.10171.
4. Le Bihan D, Poupon C, Amadon A, Lethimonnier F. Artifacts and pitfalls in diffusion MRI. Journal of Magnetic Resonance Imaging. 2006;24(3):478-88. doi:10.1002/jmri.20683.
5. Fonov V, Evans AC, Botteron K, Almli CR, McKinstry RC, Collins DL et al. Unbiased average age-appropriate atlases for pediatric studies. Neuroimage. 2011;54(1):313-27. doi:10.1016/j.neuroimage.2010.07.033.
6. Fonov VS, Evans AC, McKinstry RC, Almli CR, Collins DL. Unbiased nonlinear average age-appropriate brain templates from birth to adulthood. NeuroImage. 2009;47:S102. doi:10.1016/s1053-8119(09)70884-5.
7. Yeh FC, Tseng WY. NTU-90: a high angular resolution brain atlas constructed by q-space diffeomorphic reconstruction. Neuroimage. 2011;58(1):91-9. doi:10.1016/j.neuroimage.2011.06.021.
8. Yeh FC, Verstynen TD, Wang Y, Fernandez-Miranda JC, Tseng WY. Deterministic diffusion fiber tracking improved by quantitative anisotropy. PloS one. 2013;8(11):e80713. doi:10.1371/journal.pone.0080713.
9. Yeh FC, Wedeen VJ, Tseng WY. Generalized q-sampling imaging. IEEE transactions on medical imaging. 2010;29(9):1626-35. doi:10.1109/TMI.2010.2045126.
10. Wakana S, Caprihan A, Panzenboeck MM, Fallon JH, Perry M, Gollub RL et al. Reproducibility of quantitative tractography methods applied to cerebral white matter. Neuroimage. 2007;36(3):630-44. doi:10.1016/j.neuroimage.2007.02.049.
11. Schmidt MA, Mennecke A, Michelson G, Doerfler A, Engelhorn T. DTI analysis in patients with primary open-angle glaucoma: impact of registration on Voxel-Wise statistics. PloS one. 2014;9(6):e99344. doi:10.1371/journal.pone.0099344.
